# Beer PIMs and GEMS-Rx Prescribing in Treat-and-Release ED Visits Among Older Veterans: A Proof-of-Concept Study

**DOI:** 10.1093/geroni/igaf122.3889

**Published:** 2025-12-31

**Authors:** Michael Zanchelli, Kimberly Judon, Orna Intrator, William Hung, Ula Hwang

**Affiliations:** James J. Peters VAMC, Bronx, New York, United States; James J. Peters VA Medical Center, Bronx, New York, United States; James J Peters VA Medical Center, New York, New York, United States; James J. Peters Department of Veterans Affairs Medical Center, New York, New York, United States; NYU Grossman School of Medicine, New York, New York, United States

## Abstract

Nearly half of older Veterans (≥65 years) visiting emergency departments (EDs) receive new medications. Potentially inappropriate medication (PIMs) lists, such as Beers Criteria, primarily address long-term use, yet consideration of indications, dosing, and shorter therapies with ED prescribing may not apply.  The Geriatrics Emergency Medicine Safety Recommendations (GEMS-Rx) is a Beers subset of high-risk ED medications that include first-generation antihistamines, first-generation antipsychotics, benzodiazepines, barbiturates, hypnotics (“Z-Drugs”), metoclopramide, skeletal muscle relaxants, and long-acting sulfonylureas. This proof-of-concept study used a convenience sample of Veterans to provide initial understanding of GEMS-Rx use in VA EDs. We conducted retrospective analysis used VA’s Corporate Data Warehouse (CDW) for Veterans who received care in 12 VA Hospital-In-Home (HIH) programs between 10/2021 and 3/2024 and among them any presentation to the ED during this period. Data included patient demographics, ED visit details, and medications prescribed from the ED. Among 20,034 ED visits by 3,684 unique Veterans, mean age was 76 years ± SD 7.7, 60% were non-Hispanic White, 12% were non-Hispanic Black, 5% were female. Of these, 7,112 (35%) of the ED visits had at least one ED medication prescribed. 2,450 (34%) of these ED prescribed medications were categorized as Beers PIMs. 1,674 (68%) of the Beers PIMS were considered high-risk ED medications according to the GEMS-Rx criteria. Many ED medications prescribed to older Veterans are considered potentially inappropriate or even high-risk, underscoring gaps in age-specific medication safety. Incorporating ED-focused guidance such as GEMS-Rx could promote safer medication selection and potentially reduce adverse events.

